# The Gut Microbiota Affects Host Pathophysiology as an Endocrine Organ: A Focus on Cardiovascular Disease

**DOI:** 10.3390/nu12010079

**Published:** 2019-12-27

**Authors:** Marco Busnelli, Stefano Manzini, Giulia Chiesa

**Affiliations:** Department of Pharmacological and Biomolecular Sciences, Università degli Studi di Milano, 20133 Milano, Italy; stefano.manzini@gmail.com

**Keywords:** gut microbiota, cardiovascular disease, atherosclerosis, SCFA, bile acids, neurohormones, hormones, metabolism

## Abstract

It is widely recognized that the microorganisms inhabiting our gastrointestinal tract—the gut microbiota—deeply affect the pathophysiology of the host. Gut microbiota composition is mostly modulated by diet, and gut microorganisms communicate with the different organs and tissues of the human host by synthesizing hormones and regulating their release. Herein, we will provide an updated review on the most important classes of gut microbiota-derived hormones and their sensing by host receptors, critically discussing their impact on host physiology. Additionally, the debated interplay between microbial hormones and the development of cardiovascular disease will be thoroughly analysed and discussed.

## 1. Introduction

Humans have evolved to harbour trillions of microorganisms on and within their body. Consortia made up of bacteria, fungi, protozoa, and viruses inhabit several areas of the body such as the lungs [1], urinary tract [2], vagina [3], and skin [4], although the largest microbial community is found in the intestine [5]. The gut microbiota is mainly composed of bacteria and the dominant phyla are *Bacteroidetes*, *Firmicutes*, *Actinobacteria*, *Proteobacteria*, and *Verrucomicrobia* [3].

**Figure 1:**
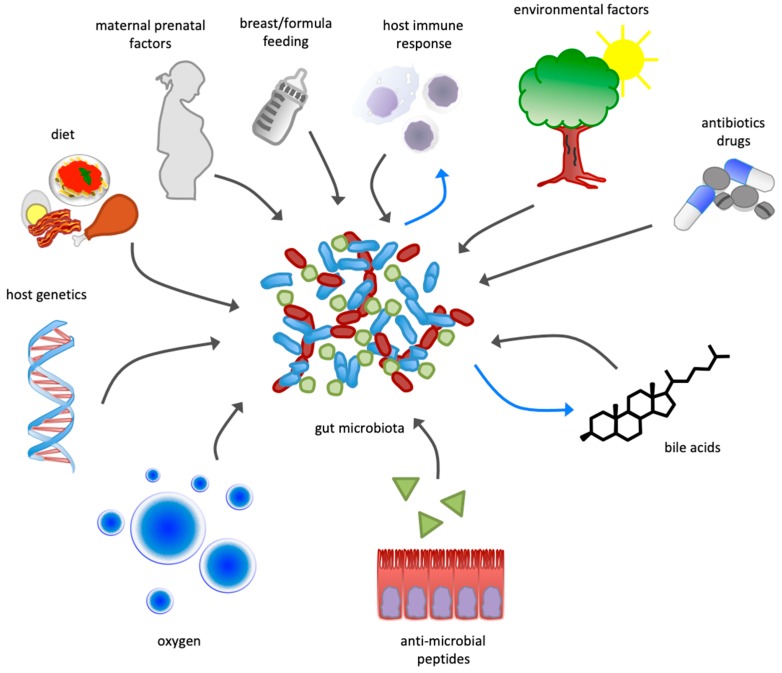
The composition of gut microbiota is influenced by exogenous and endogenous factors. While diet and host genetics have the largest influence on the makeup of microbiota, several other factors contribute to the selection of the bacterial species that live within the human gut (grey arrows). In turn, the gut microbiota is able to interact and influence some of those factors (blue arrows).

The composition of gut microbiota and the relative amount of these bacterial phyla can be modulated by endogenous and exogenous factors [6,7], including maternal prenatal factors [8], delivery mode [9], host genetics [10,11,12], host immune response [13], breast- or formula-feeding [14], dietary habits [15,16], the administration of antibiotics and other drugs [17,18], and environmental exposure [19] (Figure 1).

Additionally, each bacterial species can be further divided into clearly defined, discrete subspecies, or into a gamut of micro-organisms shaped by continuous microbial genetic variations [20]. Within the small intestine, the bacterial density is limited by the presence of oxygen [21], bile acids [22], and antimicrobial peptides [23], whereas in the large intestine, more favorable conditions—including a lower concentration of antimicrobials and a slower transit time—determine the highest bacterial load of the body [5].

The past 15 years have greatly increased our knowledge of microbial genes (the microbiome) and products, thanks to culture-independent “omic” techniques [24]. Nowadays, it is commonly believed that the metabolic capacity of the gut microbiota largely exceeds that of the human host: this assumption stems from a catalogue of 10 million non-redundant microbial genes (vs. 30,000 human genes) identified from over 1200 human faecal microbiomes collected in Europe, the United States, and China [25]. Although the biological function of the majority of bacterial gene products remains poorly defined, it has been widely demonstrated that the gut microbiota can influence the host in many ways: it modulates the immune defence [26] and provides protection against pathogens [27]; metabolizes bile acids (BAs) and xenobiotics [28,29]; it regulates not only intestinal homeostasis [30,31], but also brain function [32] and bone density [33]; it synthesizes amino acids, short chain fatty acids (SCFA) and vitamins [34]. The gut microbiota is also well-recognized for its role in regulating the metabolism of the host [35,36,37,38,39,40].

**Figure 2:**
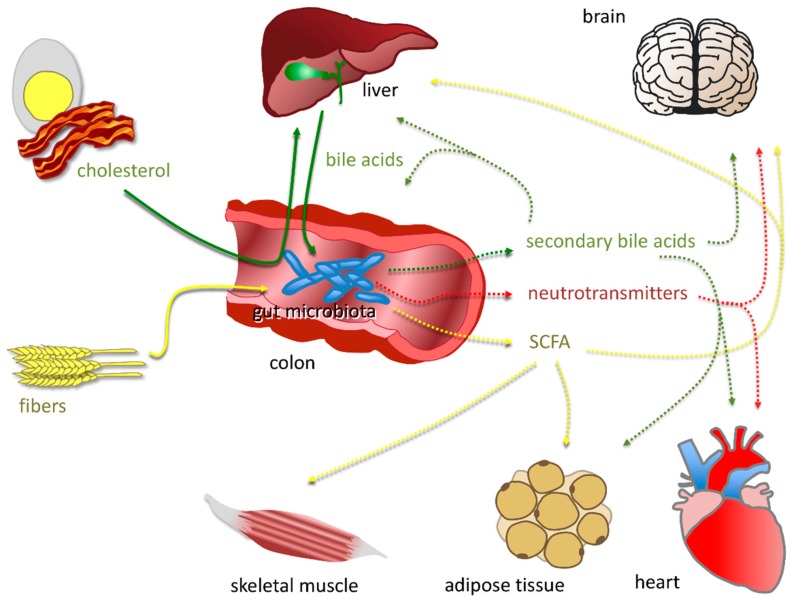
Dietary components are metabolized by gut microbiota into molecules with hormone-like properties. Dietary fibers (yellow) are metabolized into short chain fatty acids (SCFA), mainly composed of acetate, propionate and butyrate, which exert long-range effects on skeletal muscles, adipose tissue, and the liver and brain. Bile acids, first synthesized by the liver from endogenous or dietary cholesterol (green), are metabolized into secondary bile acids, acting on the intestinal wall, liver, brain, adipose tissue heart. The gut microbiota is also able to synthesize neurotransmitters (red) and neurotransmitter precursors that exert central effects. Solid line: dietary or bodily origin. Dotted line: compounds synthesized or transformed by gut microbiota.

Importantly, the gut microbiota not only contributes to host physiology, but, on the other side, alterations in the distribution of the species that make up the intestinal microbiota (dysbiosis) can predispose to a variety of chronic diseases, including inflammatory bowel disease [41], gastric ulcers [42], non-alcoholic fatty liver disease [43], obesity [44], metabolic syndrome [45], cancer [46], and several neurologic disorders [47]. The multiplicity of interactions between the gut microbiota and the pathophysiology of the host clearly indicates that the gut microbiota does not limit its effects to the intestinal milieu but is able to influence distant organs and pathways. Recently, it has been established that the gut microbiota can also affect the host also through hormone secretion, and by the production of metabolites with the ability to regulate hormone release [48]. In this respect, the gut microbiota can be fully considered as an endocrine organ. The enormous diversity of microbiota in terms of bacterial species allows for the production of a wide number of products with potential endocrine functions. The most investigated functions are reviewed here below (Figure 2). Since microbiota affects the host metabolism, and metabolic disorders are well-known contributors in the development of atherosclerosis and related cardiovascular diseases (CVD), the last part is focused on the endocrine role of gut microbiota in CVD.

## 2. The Gut microbiota as an Endocrine Organ

### 2.1. Neurohormones

The ability of bacteria to produce neurohormones—including catecholamines, serotonin and gamma-aminobutyric acid (GABA)—has been known for many years [49]. 

Catecholamines: considerable concentrations of catecholamines can be found in the gut lumen [50]. In vitro experiments have demonstrated that several bacterial species are sensitive to catecholamines, with Gram-negative bacteria being generally more responsive than Gram-positive bacteria. Several pathogens, such as *Escherichia coli*, *Klebsiella pneumoniae*, *Pseudomonas aeruginosa*, and *Staphylococcus aureus* improve their growth in the presence of norepinephrine (NE), primarily through increased iron acquisition [51]. Additionally, exposure to stress hormones may enhance the expression of those genes responsible for bacterial virulence [52]. 

The microbiota not only responds to catecholamines, but also plays a role in their biosynthesis and catabolism. Interestingly, the absence of bacteria in germ-free mice is associated with lower NE levels in the cecal lumen, and microbiota colonization is able to restore the cecal content of NE [50]. It is known that several bacterial strains have the capability to produce dopamine (DA) and NE [53]. Furthermore, bacterial β-glucuronidase of the gut microbiota can convert biologically inactive glucuronide-conjugated DA and NE into the biologically active free forms [53]. Based on these results, the gut microbiota seems to be a determinant of catecholamine concentration in the gut lumen. It has still to be established to which extent gut bacteria directly secrete catecholamines in vivo and/or if the microbiota can modulate catecholamine host production. Interestingly, it has been shown that the gut microbiota affects catecholamine turnover in the central nervous system: in germ-free mice, an accelerated metabolism of DA, NE and also serotonin was detected in brain, and was associated with changes in behaviour—i.e., increased motor activity and reduced anxiety [54]. 

Serotonin: serotonin (5-hydroxytryptamine) is known to regulate several physiological processes throughout the body, including neurological functions, gastrointestinal secretion and peristalsis [55,56]. Despite the relevant role of central serotoninergic mechanisms, the brain contains low relative concentrations of serotonin. The vast majority of it—about 95%—is, in fact, produced in the bowel by the enterochromaffin cells (EC), which express tryptophan hydroxylase-1 (TPH1), the rate-limiting enzyme in serotonin’s biosynthesis from the amino acid tryptophan [57]. Even though some bacterial species—such as *Escherichia coli*—are known to synthesize tryptophan, there is no evidence that the bacterial community makes a significant contribution toward its production in humans [58]. Besides serotonin production, tryptophan is processed by the microflora of the gastrointestinal tract into ligands of the aryl hydrocarbon receptors (AHRs) [59], as well as into kynurenine and related end products [60]. EC-secreted serotonin reaches the intestinal lumen and the blood, but it cannot cross the blood-brain barrier [55]. 

Several studies have demonstrated that gut microbiota can modulate host serotonin concentration. First of all, a metabolomic analysis showed that blood serotonin levels are lower in germ-free mice and are normalized after microbiota colonization [61]. Different bacteria have the capability to directly produce serotonin, although the possible relevance of this property in vivo is yet to be determined [49]. Additionally, similarly to catecholamines, serotonin undergoes deconjugation by the bacterial enzymes of the gut microbiota with the formation of the biologically-active free molecule [62]. However, the microbiota is believed to influence host serotonin levels, mostly through the secretion of SCFA or BAs (see below), which stimulate the EC to produce serotonin, specifically by increasing the expression of TPH1 [63,64].

Gamma-aminobutyric acid (GABA): GABA is an inhibitory neurotransmitter playing a role not only in the central nervous system, but also in the control of several processes occurring in the gut, including the regulation of intestinal motility, gastric emptying and acid secretion [65].

The microbiota seems to affect peripheral GABA levels, as shown in germ-free animals, where GABA concentrations in colonic lumen and plasma were lower—whereas cerebral concentrations were unchanged—compared with those of colonized mice [66].

Gut bacteria have a strong, bidirectional relationship with GABA. GABA production, originating from the decarboxylation of glutamate, has been reported for a large number of bacterial species [49] and plays a relevant role in bacterial physiology, since it contributes to the maintenance of intracellular pH homeostasis [67]. Subspecies of *Escherichia coli* can grow on GABA as sole carbon and nitrogen source [68]. Furthermore, some intestinal bacteria can only grow when other bacteria, such as *Bacteroides fragilis*, act as a helper bacterium to produce GABA—the only substrate they can grow onto [69]. Very recently, the whole genomic sequences of ~1200 bacterial species identified in the human gut were evaluated against all the known GABA-consumption and -production enzyme sequences. Approximately 100 species were found to be able to synthesize GABA, ~200 possessed the enzymatic machinery to consume it, and ~210 were able to both produce and consume it [69].

In an attempt at assessing whether the administration of probiotic bacteria could affect brain function, *Lactobacillus rhamnosus JB-1* was chronically administered to healthy mice and found to reduce depressive- and anxiety-like behaviour. Interestingly, this effect was associated with changes in the expression of cerebral GABA receptors [70]. More recently, the same research group showed, using magnetic resonance spectroscopy, that *Lactobacillus rhamnosus JB-1* treatment in mice was able to increase GABA cerebral concentration by about 25% [71]. Despite these promising experimental results, a clinical study failed to show any effect on stress and cognitive performance by *Lactobacillus rhamnosus JB-1* given to healthy male volunteers [72]. Other studies will be needed to evaluate the possible effects of probiotics on stress-related disorders in healthy or diseased subjects.

### 2.2. Bile Acids

BAs are soluble products derived from the catabolism of highly insoluble cholesterol. Notably, almost all cells are able to synthesize cholesterol from acetate or get it from the diet (~70% and ~30%, respectively), but only the liver can eliminate cholesterol via secretion into the bile, unmodified, or converted into BAs. The whole-body cholesterol balance is thus finely tuned by the interactions among cholesterol synthesis, absorption and excretion [73].

In hepatocytes, the biosynthesis of the two primary BAs, cholic acid (CA) and chenodeoxycholic acid (CDCA), occurs via two different series of enzymatic reactions: the classical pathway, initiated by the enzyme cholesterol 7a-hydroxylase (CYP7A1), and the alternative pathway initiated by sterol-27-hydroxylase (CYP27A1) [74]. The 27-hydroxycholesterol is further hydroxylated by oxysterol 7a-hydroxylase (CYP7B1). The CA:CDCA ratio is determined by the sterol 12a-hydroxylase (CYP8B1), which is required for CA synthesis [75]. Interestingly, the expression of CYP7A1, CYP7B1, and CYP27A1, but not that of CYP8B1, is regulated by gut bacteria [76].

CA and CDCA are then extensively conjugated in a two-step process to either glycine (75%) or taurine (25%), transported into bile via the bile salt export pump (BSEP), and subsequently stored in the gallbladder until their release into the duodenum after the ingestion of a meal [28]. The conjugation process increases the water-solubility and detergent properties of BAs, thus facilitating the emulsification and absorption of dietary cholesterol, triglycerides and fat-soluble vitamins [77].

Over 90% of conjugated BAs are reabsorbed in distal ileum via the apical sodium-dependent bile acid transporter (ASBT or SLC10A2) located on the enterocyte brush border. Once in the enterocyte, BAs are transported to the basolateral membrane by the intestinal bile acid-binding protein (IBABP) and then effluxed by OST-α/β into the blood to be recirculated via the portal vein to the liver [74,78].

The bacterial metabolism of the BAs that are not reabsorbed represents one of the most intriguing relationships linking gut microbiota to host physiology. Microbial deconjugation removes glycine or taurine from the sterol core of the primary BAs, generating less soluble, unconjugated BAs and prevents their ASBT-mediated reuptake from the small intestine, thus increasing the excretion of free BAs into faeces [79]. The deconjugation is catalyzed by bile salt hydrolases (BSHs), enzymes that are expressed by numerous strains belonging to several bacterial genera, including the Gram-positive *Bifidobacterium*, *Lactobacillus*, *Clostridium* and *Enterococcus* and the Gram-negative *Bacteroides* [80,81,82]. 

Because bile acids are toxic molecules to bacteria due to their acidic nature and detergent-like properties [83], it has been hypothesized that the wide distribution of BSHs may indicate that BAs deconjugation facilitates the symbiosis between gut bacteria and the human host [84,85]. In addition, it has been proposed that the released glycine and taurine could represent a source of nutrients for bacteria, including glycine as an energy, carbon, and nitrogen source, and taurine as a sulphur source [79].

A small number of deconjugated secondary bile acids can be absorbed from the gut through passive diffusion, joining the enterohepatic circulation and possibly acting as signaling molecules in the host. Deconjugated primary bile acids descend to the colon, where they can be 7-dehydroxylated, and oxidized/epimerized by the intestinal microbiota, markedly increasing the diversity of BAs (Table 1) [86].

The 7-dehydroxylation generates the secondary BAs lithocholic acid (LCA) from CDCA and deoxycholic acid (DCA) from CA in humans. Although LCA and DCA are absorbed to some extent, 7-dehydroxylation is the most important bacterial biotransformation, with LCA and DCA being the two predominant bile acids in human faeces [79,87]. Bacteria able to perform this reaction belong to the *Clostridium* (clusters XIVa and XI) and *Eubacterium* genera, both included in the *Firmicutes* phylum [79,88,89,90].

The oxidation and epimerization of the 3-, 7-, or 12-hydroxyl groups of BAs are carried out by the hydroxysteroid dehydrogenases (HSDHs), which were found in *Actinobacteria*, *Proteobacteria*, *Firmicutes*, and *Bacteroidetes* [91,92,93,94,95]. It has been hypothesized that bacteria epimerize BAs to make them less toxic and harmful to bacterial membranes [73]. In rodents, the secondary bile acids generated by the activity of gut microbiota are murideoxycholic acid (MDCA), omega-MCA (ωMCA), hyodeoxycholic acid (HDCA), and hyocholic acid (HCA).

Germ-free mice have larger gallbladders and higher levels of BAs than those of conventionally raised mice, with a differential bile acid profile characterized by an increased prevalence of conjugated primary BAs and absence of secondary BAs [96].

The fraction of BAs that is not reabsorbed into enterohepatic circulation and not excreted with the faeces, can enter the systemic circulation through transcellular passive or active mechanisms [97]. 

Circulating BAs act as signaling molecules able to regulate several processes such as their own synthesis [98], glucose and energy homeostasis [99,100,101], intestinal peristalsis [102], inflammation [103,104], gut microbiota composition [105], and skeletal muscle mass [106]. 

The systemic signaling activity of BAs is primarily mediated by two receptors, the farnesoid X receptor (FXR) and the Takeda G protein-coupled receptor 5 (TGR5) (Table 1) [107,108,109]. Additionally, the pregnane X receptor (PXR) [110], the sphingosine-1-phosphate receptor 2 (S1PR2) [111], and the vitamin D receptor (VDR) [112], can be activated by BAs or their metabolites.

FXR is widely expressed, but plays a pivotal role in liver, ileum [113,114], kidney [115], white adipose tissue (WAT) [116] and heart [117].

In the liver, bile acid-activated FXR forms a heterodimer with retinoic X receptor (RXR) and suppresses the expression of CYP7A1, thus reducing the hepatic conversion of cholesterol into BAs [108,118]. At the same time, in the ileum, FXR induces the expression of circulating fibroblast growth factor 19 (FGF19); FGF19 reaches the liver with portal blood, where it binds to the FGF receptor 4 and inhibits the expression of both CYP7A1 and CYP8B1, further reducing the synthesis of BAs [108,119].

In addition, as mentioned above, the composition of gut microbiota can be deeply influenced by BAs and when the bile flow into the intestine is hampered, bacterial overgrowth and translocation of bacteria in the small intestine occur [120,121]. Interestingly, in addition to their detergent properties, BAs can prevent bacterial growth via the FXR-mediated transcription of antimicrobial agents [122].

TGR5 is ubiquitously expressed with high expression in nonparenchymal cells of the liver [123], intestinal enteroendocrine cells that secrete the incretin hormone glucagon-like peptide-1 (GLP-1), enteric neurons [123,124], placenta, lung, spleen, WAT, brown adipose tissue, skeletal muscle, and bone marrow [125,126,127]. TGR5 is mainly activated by the BAs LCA and DCA [126,128]. In enteroendocrine L cells, TGR5 activation promotes the release of GLP-1, whereas in brown adipose tissue and skeletal muscle, it promotes energy expenditure via the augmented activity of type-2 iodothyronine-deiodinase which, in turn, upregulates the expression of uncoupling proteins 1 and 3 (UCP1 and UCP3) [109]. Taken together, the effects described above improve glucose metabolism and insulin sensitivity [99,109]. In support of these findings, TGR5^-/-^ mice display impaired glucose tolerance, whereas TGR5-overexpressing mice display enhanced GLP-1 secretion and insulin release in response to a glucose load [99].

PXR is a nuclear receptor that acts as a xenobiotic sensor. It is highly expressed in the liver and intestine, where it modulates the expression of several cytochromes. PXR can be activated by the toxic bile acid LCA, but only at supraphysiological concentrations, such as those that take place during severe cholestasis [129].

VDR is expressed at high levels in the small intestine, kidneys, osteoblasts and many types of immune cells. Similar to PXR, also VDR is also activated by LCA as well as LCA-derived metabolites and regulates the expression of genes involved in bile acid synthesis, conjugation, transport and metabolism.

S1PR2 is highly expressed in hepatocytes where it can be activated by taurocholate (TCA) and other conjugated BAs, but not unconjugated BAs. The binding of conjugated BAs to S1PR2 increases sphingosine-1-phosphate (S1P) biosynthesis, while reducing ceramide, sphingomyelin and glucosylceramide concentration [111]. In addition, bile-acid activated S1PR2 determines insulin-like activity in the hepatic glucose metabolism, with a downregulation of the gluconeogenesis genes, PEP carboxykinase (PEPCK) and glucose-6-phosphatase (G6Pase) [130].

### 2.3. Short Chain Fatty Acids

The human gut is able to digest and absorb many nutrients present in food, but several carbohydrates, the so-called dietary fibres, escape digestion in the upper gastrointestinal tract and can be fermented by the anaerobic microbial community in the caecum and colon [131]. Those include structural non-starch polysaccharides, resistant starch and some oligosaccharides [132]. 

The most abundant metabolites generated by the gut microbiota from the breakdown of undigestible carbohydrates are SCFA, defined as the fatty acids with less than six carbons, thus spanning from formic to valeric acid. Among them, acetic, propionic and butyric acid account for 95% of the whole SCFA. They are generally found in the colon in a ratio of 60/20/20, and their combined concentration ranges from 50 to 150 mM [133]. Acetate is the most abundant SCFA, being a fermentation product from pyruvate via acetyl-CoA of most anaerobic bacteria as well as being produced by acetogenic bacteria through other pathways [134]. Propionate and butyrate are instead produced by different gut bacteria. Specifically, propionate is mainly formed by *Bacteroidetes* as well as by the *Negativicutes* class of *Firmicutes* [135], whereas butyrate is produced by several species belonging to *Ruminococcaceae* and *Lachnospiraceae*, as well as to other families of human colonic *Firmicutes* [136,137]. 

After their production by the microbiota, SCFA are almost completely absorbed by colonic cells through active transport, passive diffusion or in exchange with bicarbonate. Reportedly, not only the production rate of SCFA varies throughout the large intestine [138], but also their absorption. For instance, it has been shown that acetate absorption peaks in the distal colon [139]. Active transport is the main route of SCFA absorption and is mediated by monocarboxylate transporters (MCT). Specifically, MCT1 transports SCFA in a H^+^-dependent manner, whereas a sodium-dependent MCT1 (SMCT1 or SLC5A8) transports SCFA anions [140]. 

After absorption, SCFA and particularly butyrate, are partially used as energy sources by the colonocytes, and the fraction not metabolized is transported into portal circulation [141,142]. In the liver, besides the use of SCFA as energy source, acetate is precursor for the synthesis of cholesterol and fatty acids, and propionate contributes to gluconeogenesis [143]. The end result is that only about 40%, 10%, and 5% of the acetate, propionate and butyrate, respectively, produced by the microbiota reaches the systemic circulation. Plasma concentrations have been reported to be in the range 25–250 μM for acetate, 1.4–13.4 μM for propionate, and 0.5–14.2 μM for butyrate [138,144]. 

SCFA exert several beneficial effects for gut health. SCFA, particularly butyrate, contribute to the maintenance of barrier integrity by regulating the expression of tight junction proteins [145,146]. Additionally, acetate and butyrate stimulate mucin production in the gastrointestinal tract [147]. Moreover, SCFA can affect gastrointestinal motility through several mechanisms, including the release of the gut hormone peptide YY (PYY) from enteroendocrine L-cells [148] and SCFA-induced serotonin release from enterochromaffin cells (EC) [149]. SCFA can also influence appetite and food intake by regulating not only PYY, but also leptin and ghrelin production, through mechanisms still not clarified [150]. Glucose metabolism is also affected by SCFA, which have been shown to modulate the plasma levels of insulin and GLP-1 [151]. Finally, SCFA and particularly butyrate mediate several inhibitory effects on tumorigenesis, including decreased proliferation of cancer cells, anti-inflammatory and immunomodulatory activities, thus possibly reducing the risk of colorectal cancer [133,152]. SCFA are able to modulate biological responses of the host also in other organs including the brain, as it was shown that SCFA can cross the blood-brain barrier [153]. 

Many of the effects mediated by SCFA occur through two major mechanisms: (a) a direct inhibition of histone deacetylases (HDAC) and the consequent regulation of gene expression; (b) signalling through orphan G protein-coupled receptors (GPR). 

Acetylated histones have a less compact and more transcriptionally active chromatin, whereas the removal of acetyl residues leads to a transcriptionally silenced chromatin [154]. HDAC remove acetyl groups from histones; therefore, the inhibition of HDAC activity or expression can increase gene transcription [154,155]. Several studies have demonstrated that SCFA inhibit HDAC activity, thus they can alter gene expression in a wide variety of cells. SCFA are believed to exert this effect by direct interaction with HDAC after entrance into the cells [156], or indirectly, through GPR signalling [157]. Of all the SCFA, butyrate is considered the most potent inhibitor of HDAC activity, but also propionate and acetate can also affect histone acetylation, depending on the tissue and the cell type considered [158,159].

SCFA-mediated HDAC inhibition results in an anti-inflammatory immune phenotype [160]. SCFA decrease the production of pro-inflammatory cytokines in macrophages [161] and peripheral blood mononuclear cells [162] through the modulation of NF-kB [162]. SCFA, specifically butyrate, was also shown to play a role in the suppression of inflammatory and allergic responses by inducing the differentiation of regulatory T cells [163]. These results provide a molecular insight into the therapeutic use of butyrate to ameliorate chronic inflammatory conditions, such as inflammatory bowel disease (IBD) [134]. Moreover, butyrate may inhibit proliferation and induce apoptosis in colon cancer cell lines [164].

SCFA-mediated HDAC inhibition also affects brain functions, such as behaviour, learning and memory [150]. However, the dosage of SCFA seems to be critical to obtain such an effect, thus accurate dose-response investigations will be required to confirm these observations.

SCFA are also involved in several interactions with the surface-exposed receptors of host cells. The most investigated SCFA receptors are GPR41 also named free fatty acid receptor 3 (FFAR3 or FFA3), GPR43 (FFAR2 or FFA2), and GPR109A. GPR43 recognizes all the three major SCFA [165], whereas the affinity for GPR41 is higher for propionate than butyrate and low for acetate [166], and GPR109A mainly interacts with butyrate [167]. GPR43 is expressed along the entire gastrointestinal tract, mainly in the enteroendocrine L-cells producing PYY and GLP-1 [168,169], the gut hormones involved in gut motility, satiety and glucose metabolism. SFCA have been shown to activate GPR43 in vitro, and mice deficient in GPR43 show a reduced SCFA-stimulated release of GLP-1, together with impaired glucose tolerance [169]. GPR43 is also expressed in WAT [170,171] and its deletion in mice results in obesity, whereas the adipose-specific overexpression of GPR43 is associated with a lean phenotype [172]. Finally, GPR43 is also expressed in granulocytes, monocytes, dendritic cells and mast cells [173,174,175], indicating the role of SCFA in inflammatory/immune response. Indeed GPR43^-/-^ mice exhibit exacerbated or non-resolving inflammation in models of colitis, arthritis and asthma [176].

GPR41 is expressed in intestinal enteroendocrine L-cells, where it regulates the secretion of PYY and GLP-1, and it is also found in adipose tissue, the pancreas, spleen, lymph nodes, bone marrow, and peripheral blood mononuclear cells including monocytes [165,173]. GPR41 is also expressed in the sympathetic ganglia and its activation leads to the release of NE by sympathetic neurons [177] with a consequent increase in energy expenditure.

GPR109A is expressed in the epithelial cells of the colon, adipose tissue and inflammatory cells. GPR109A might be involved in the anti-inflammatory/tumor suppressor actions of butyrate, by stimulating the differentiation of regulatory and IL-10-producing T cells [167], by suppressing the activation of nuclear factor-κB (NF-κB) and by inducing apoptosis independent of HDAC inhibition [178]. 

## 3. Effects of Gut Microbiota-Derived Metabolites on Atherosclerosis and Cardiovascular Risk Factors

Accumulating data indicate that a high-fat, high-cholesterol diet aggravates cardiovascular disease; at the same time, several population-based studies have revealed that diet is a strong modulator of gut microbiota [179], and that permanent changes in microbiota composition might be achieved through dietary modifications [36,180]. In humans, diets rich in fat and protein correlate with an increased abundance of *Bacteroides*, whereas high-fiber diets correlate with increased bacterial richness and an abundance of *Prevotella* [16,181,182,183]. The consumption of diets composed entirely of animal products triggers enrichment in bile tolerant bacteria (*Bacteroides*, *Alistipes,* and *Bilophila*) and a depletion in *Firmicutes* able to metabolize plant polysaccharides (*Roseburia*, *Eubacterium rectale,* and *Ruminococcus bromii*) [15]. In mice, the consumption of the so-called “Western diets”, low in fibers and enriched in total fat, animal proteins and refined sugars, associates with a decrease in *Bacteroidetes* levels and an increase in *Firmicutes* and *Proteobacteria* [184,185]. Similarly, the microbiota of children from a rural African village shows a significant enrichment in *Bacteroidetes* and depletion in *Firmicutes*, mainly from the genus *Prevotella* and *Xylanibacter*, compared with Italian children, whose diet is characterized by a paucity of dietary fibre and who harbour increased levels of *Enterobacteriaceae*, predominantly *Shigella* and *Escherichia* [16].

Moreover, dietary fibers stimulate mucus production from the intestinal epithelium [186], and the dietary deficiency of fibers damages the mucus barrier being associated with an increased presence of mucin-degrading bacteria (*Akkermansia muciniphila* and *Bacteroides caccae*) and a concomitantly reduced presence of fibre-degrading species (*Bacteroides ovatus* and *Eubacterium rectale*) [187]. Consistent with this, the administration to mice of a Western diet, low in fiber content, increases the penetrability of the inner mucus layer and renders it penetrable, thus increasing the susceptibility to infections [188].

To make matters even more complicated, previous observations have demonstrated that conventionally raised ApoE^-/-^ mice fed a low-cholesterol diet have reduced aortic plaques compared to germ-free ApoE^-/-^ mice [189,190]. Conversely, after the administration of a Western diet, atherosclerosis development in conventionally raised ApoE^-/-^ mice was comparable to that in germ-free ApoE^-/-^ mice [190,191]. Thus, it can be hypothesized that the gut microbiota could have an impact on atherosclerosis development through the production of some of the metabolites/hormones discussed above [192].

### 3.1. Neurohormones and CVD

The role of catecholamines in the cardiovascular system is very well-known and, recently, has been extensively reviewed [193]. Catecholamines mediate the activation of the sympathetic nervous system and, through the binding to specific receptors, regulate the vascular tone [194] as well as heart rate and contractility [195]. The high relevance of catecholamines in cardiovascular health and disease is demonstrated by the huge number of drugs that, acting on their receptors, regulate blood pressure and heart function [196]. The actual literature does not provide sufficient evidence of the possible ability of microbiota to affect peripheral catecholamine levels in such a way to influence cardiovascular function. However, the proven ability of microorganisms to synthesize catecholamines or to interfere with their metabolism suggests the need for further investigations in this direction. 

As mentioned above, serotonin is unable to cross the blood-brain barrier. Peripherally, it is mostly synthesized in EC cells by the enzyme TPH1, whereas TPH2 regulates serotonin central production [197]. The pathophysiology of peripheral serotonin has been extensively studied in mice lacking TPH1. TPH1 deficiency in mice does not alter serotonin levels in the brain, but it does result in very low circulating serotonin concentrations [57]. When mice are maintained on high fat diet, lack of TPH1 results in lower body weight, less adiposity [198,199] and the reduced expression of markers of adipose tissue inflammation [200], compared with wild-type mice. Additionally, TPH1-deficient mice display lower lipid accumulation in liver and improved glucose tolerance and insulin sensitivity [198,199]. TPH1 deficiency also deprives platelets of their serotonin content and reduces their reactivity [201], which results in delayed thrombus formation and lower inflammatory cell recruitment [202]. All these observations suggest the benefit of low peripheral serotonin levels on metabolic disorders and thrombosis, and, as a consequence, on associated CVD. Serotonin, through its receptors, also plays a role in controlling blood pressure and in heart pathophysiology [203], being essential for a correct cardiac development [204] and potentially involved in cardiac hypertrophy [205] and heart valve disease [206]. The described studies and several other investigations have prompted the development of TPH1 inhibitors as a therapeutic option for several pathological conditions [207].

Serotonin seems to affect microbiota and SCFA concentrations, as shown in homozygous and heterozygous TPH1-deficient mice, where lower serotonin concentrations are associated with changes in microbiota composition and reduced SCFA levels [208]. On the other hand, through SCFA and secondary bile acids, the microbiota can stimulate serotonin production in EC cells, by upregulating the expression of TPH1 [63,64]. Based on the above considerations, increasing serotonin levels may have a detrimental effect on CVD. However, it should be considered that peripheral serotonin exerts a multiplicity of metabolic functions, which include serotonylation of small G proteins, an essential step for insulin secretion from the pancreatic β cells [209]. Further studies will be required to establish the effects of serotonin modulation on metabolic disease and if microbiota metabolites play a beneficial or a detrimental role in this context.

Of note, other tryptophan-derived metabolites may play a role in CVD. Kinurenine and related metabolites are involved in the regulation of inflammation and immune response. The production of these metabolites is regulated through the rate-limiting enzyme Indoleamine 2,3-dioxygenase 1 (IDO1). Interestingly, human subjects with metabolic syndrome have increased IDO1 activity with consequent higher levels of kynurenine. Furthermore, kynurenine to tryptophan ratio correlates with obesity, BMI, and blood triglyceride levels [210].

GABA is supposed to mediate athero-protective actions. Several studies have shown that GABA negatively modulates a variety of functional properties of several immune cells (macrophages, dendritic cells and T cells), such as cytokine secretion, cell proliferation, phagocytic activity and chemotaxis [211]. Specifically, treatment with GABA inhibits the formation of human macrophage–derived foam cells in vitro via a restoration of the physiological macrophage cholesterol metabolism [212]. Furthermore, an increased plasma GABA concentration ameliorates the progression of autoimmune diseases, such as multiple sclerosis, diabetes mellitus and rheumatoid arthritis [213,214]. 

An ever-growing body of research emphasizes the ability of gut microbiota-produced GABA to influence the host, and possibly to exert a beneficial effect on atherosclerosis-predisposing conditions. It has been recently demonstrated that fecal transplants from lean donors were able to raise plasma GABA levels in obese individuals [215]. Furthermore, the dietary administration of the GABA-producing *Lactobacillus brevis DPC 6108* to insulin-resistant rats improved glucose homeostasis [216].

On the other hand, as already mentioned, there are indications that germ-free mice have reduced plasma GABA levels [66,217] and this is in accordance with the fact that germ-free ApoE^-/-^ mice develop more atherosclerosis than conventional mice with the same genotype [189,190].

### 3.2. Bile Acids and CVD 

In addition to impacting the composition of gut microbiota, the consumption of a high-fat diet modifies BA profiles and results in a significantly higher excretion of fecal secondary BAs, mainly DCA and LCA [218,219]. However, the gut microbiota-mediated regulation of bile acids in atherogenesis is not fully understood, mainly because conflicting data exist on the effects of FXR and TGR5 on atherosclerosis development, several FXR- and TGR5-expressing tissues are involved, and the results obtained so far come from studies in rodents.

FXR^-/-^ mice fed a chow diet display increased plasma levels of high-density lipoprotein cholesterol (HDL-C), non-HDL-C and triglycerides, increased apolipoprotein B-containing lipoprotein synthesis, and a reduced expression of hepatic genes involved in reverse cholesterol transport compared to wild-type mice [220]. In both the atherosclerosis-prone ApoE^-/-^ and Ldlr^-/-^ mice, the activation of FXR with a synthetic agonist inhibits the diet-induced increase in non-HDL-C and triglyceridemia and determines a near complete inhibition of aortic lesion formation [221]. In accordance, FXR^-/-^ ApoE^-/-^ mice have increased blood levels of cholesterol and triglycerides, a more severe pro-atherogenic plasma lipoprotein profile, and a more severe atherosclerosis [222,223].

Conversely, the administration of a Western diet to FXR^-/-^ Ldlr^-/-^ male mice leads to a reduced atherosclerosis development as compared with Ldlr^-/-^ mice. Double knockout mice were characterized by reduced plasma levels of low-density lipoprotein cholesterol (LDL-C) and HDL-C, whereas triglyceride levels were increased. FXR^-/-^ Ldlr^-/-^ male mice also showed a reduced expression of CD36 in macrophages, a finding commonly associated with a reduction in foam cell formation and atherosclerosis [224].

Likewise, the activation of TGR5 by a TGR5-specific agonist significantly reduces atherosclerosis formation in Ldlr^-/-^ mice, mainly through reduced macrophage activation and the production of pro-inflammatory cytokines as the direct result of the NF-κB pathway blockade. Contrastingly, the lack of TGR5 in Tgr5^-/-^ Ldlr^-/-^ mice does not significantly worsen atherosclerosis as compared with Ldlr^-/-^ mice [225].

Taken as a whole, the results seem to suggest that the loss of one receptor is compensated by the presence of the other. In this respect, the simultaneous deficiency of FXR and TGR5 dramatically worsens atherosclerosis development and aortic inflammation in Ldlr^-/-^ mice fed a high-fat diet [226], whereas the administration of a potent dual activator for FXR and TGR5 significantly reduces atherosclerotic plaque formation [227].

**Figure 3:**
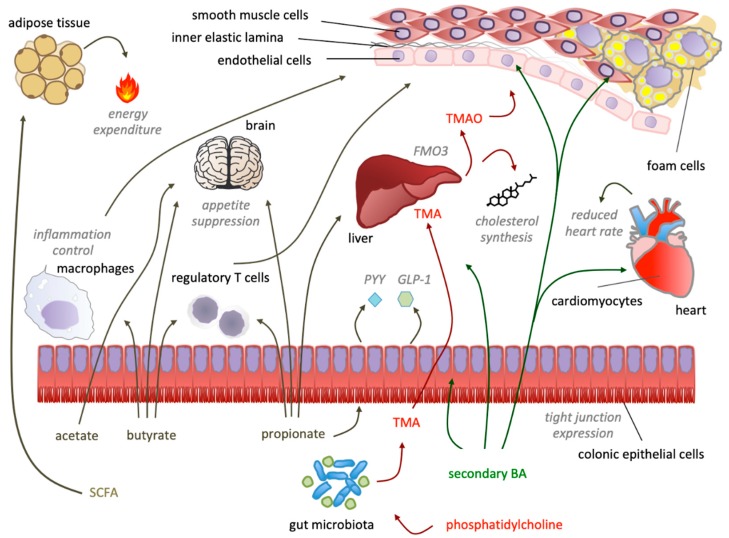
The gut microbiota synthesizes hormone-like molecules that can influence the development of cardiovascular disease. SCFA produced by microbiota (dark yellow) have pleiotropic effects that modulate gut endothelial cells permeability, inflammatory cells activation, glucose tolerance and satiety—ultimately impacting on cardiovascular disease. Hepatic (primary) bile acids are metabolized in the gut into secondary bile acids, that act as long-range signaling molecules (dark green) with effects on heart rate, cholesterol synthesis and gut epithelium integrity. The metabolism of choline-containing compounds supplies the liver with trimethylamine (TMA, dark red), which is oxidized by hepatic FMO3 into TMAO, exacerbating atherosclerosis development.

Bile acid receptors are also expressed on endothelial cells, vascular smooth muscle cells and cardiomyocytes, with resulting additional cardiovascular effects [228,229,230,231]. In this respect, BAs have been shown to induce negative chronotropic effects on cardiomyocytes [229,232], and to reduce blood pressure [233], mostly mediating vasorelaxation [234,235]. Of note, pinning the specific function of BAs to specific receptors has been proven difficult, given the breadth of downstream function exerted by the activations of many diverse receptors, further hampered by the vastly different chemical properties of various BAs [236]. Differences in hydrophobicity and conjugation state, for example, result in different amplitudes of vasodilatory effects (Figure 3) [237].

### 3.3. SCFA and CVD

Dietary fibers have been considered among the approaches to help reduce obesity, as they promote satiety [238]. Indeed, the water binding capacity, and more importantly, the fermentability of the fibers by gut microbiota to SCFA is directly associated with a reduction of meal numbers and an increase in inter-meal intervals.

Butyrate-producing bacteria are pivotal modulators in the regulation of inflammation, both locally within the intestine, and on its peripheries. Butyrate is particularly important for the regulation of the intestinal homeostasis of macrophages [239], as well as the regulation of the intestinal barrier function [240,241]. This can be the direct consequence of the metabolism of colonic epithelial cells, which is mainly dependent on butyrate and fatty acid oxidation: the resulting slight hypoxia can in turn induce the expression of hypoxia inducible factor 1 (HIF-1a), which—among other functions—upregulates the expression of tight junction genes [242]. Alternatively, or additionally, the histone deacetylase activity displayed by butyrate has been shown to upregulate the expression of tight junction genes, as well as that of intestinal mucins and cytokines [243,244,245].

The capability to modulate inflammation can partly explain the protective effects that butyrate exerts in IBD [246], even though it has been very recently demonstrated that this is highly dependent upon the context of both the microbiota and fiber composition, as butyrate-producing bacteria can, in susceptible hosts, exacerbate inflammation [247]. Systemically, butyrate orchestrates the modulation of inflammation in metabolic syndrome-related pathologies. Butyrate-producing bacteria are reduced in diabetes [248], and butyrate supplementation is able to reduce diabetic inflammation [249]. In addition to the aforementioned effects on satiety, it is also believed that butyrate can enhance energy expenditure, thus reducing obesity, by activating β3-receptor-mediated lipolysis in WAT [250]. Butyrate-producing bacteria have recently been found to be capable of reducing atherosclerosis [251,252]. Opposing the effects described below of the bacterial conversion of TMA into pro-atherogenic TMAO, *Roseburia* and other butyrate-producing bacteria correlate with reduced lesion sizes in ApoE^-/-^ mice. Experiments performed in germ-free ApoE^-/-^ mice colonized with defined communities demonstrate how *Roseburia intestinalis* is able to ferment a high-fiber-content diet into butyrate, resulting in four-fold higher levels of butyrate in the caecum, but—interestingly—not in plasma, and in reducing the extent of plaque. The same effects are not reproduced without *Roseburia intestinalis* and can only be attained through an exogenous butyrate source [251,252]. What links butyrate production to reduced plaque size—since its concentration in plasma is unchanged as well as those of cholesterol and other lipids—is, ultimately, the (already reviewed) positive effect of butyrate on the gut barrier function and macrophages. The decreased permeability of the intestinal wall to LPS, resulting in a decreased systemic inflammation, ultimately leads to a decreased expression of pro-inflammatory cytokines, resulting in a reduction of plaque.

Even though some paradoxical effects of butyrate, such as the ones regarding IBD, have been previously reported, and butyrate might itself be a double-edged sword [253], it is generally assumed that an increase in butyrate-producing bacterial species might result in overall positive effects. It has been proposed that a way to stimulate butyrate production might rely on the dietary supplementation of carbohydrates that resist digestion by human enzymes, but can be subsequently metabolized by colonic bacteria. This has largely proven to be an oversimplification, as it is heavily dependent on the makeup of the microbiota, and fiber degradation is not a sure way to lead towards butyrate production [254]. Trying to identify combinations of butyrogenic bacteria that can attack a broad range of substrates is challenging, as many bacteria are specialized in metabolizing specific bonds: to improve the efficacy of dietary supplements, a personal treatment tailored to each individual microbiota might be necessary [254].

Propionate, from a nutritional standpoint, is unique among SCFA, as it can be incorporated into glucose via gluconeogenesis, owing to its odd number of carbon atoms. However, the incorporation of colonic-derived propionate into glucose is as low as 5.9% in humans, compared to the much higher value of 62% of mice [255]. Very recently, a study found that propionate treatment in ApoE^-/-^ mice resulted in a broad range of positive effects [256]. The area of aortic lesions was reduced in mice receiving propionate in drinking water—an effect that was dependent on the activity of regulatory T cells, as demonstrated by their depletion. Contrary to butyrate—the preferred energy source of colonocytes, which hampers its passage into the bloodstream, and whose distant actions are not necessarily mediated by its increase in the plasma—propionate and acetate can freely travel across the epithelium and reach the liver [257,258]. Even if only small amounts of propionate can be found in circulation [257], local effects can also be ascribed to propionate, for example the tightening of the intestinal epithelial cells via the induction of the expression of tight junctions [259]. Similar to butyrate, propionate does appear to regulate inflammation with hormone-like properties.

The beneficial effects of propionate may also be partly dependent on a cholesterol lowering activity. There are several reports in the literature where, spanning a period of at least 40 years, at least in the animal model, propionate has been shown to display lipid- and cholesterol- lowering activities. First, dietary fibers were associated with these findings [260], eventually pinning the effect down to propionate in a rat model, where in liver reperfusion experiments propionate was shown to directly inhibit cholesterol synthesis [261]. The literature is littered with similar reports, with recent research making the connection between particular bacterial strains, propionate production and reduced plasma cholesterol levels [262]. These findings do not, however, seem to be translatable into a human setting. The dietary supplementation of 10 g of sodium propionate was unable to change the lipid metabolism of six healthy volunteers [263]. This can be explained taking into account the diverse response of rat and human hepatocytes: in one in vitro study, propionate was found to inhibit lipid synthesis in rat when given a dose of 0.1 mM, whereas a similar effect was only attained with human hepatocytes with 200 times the propionate concentration—as high as 20 mM [264]. The jury is still out on propionate regulation of lipid metabolism: the clinical trial Safety and Efficacy of Propionate for Reduction of LDL Cholesterol (PROPER-LDL) (https://clinicaltrials.gov/ct2/show/NCT03590496), which aims at assessing the effects of a daily supplement of 500 mg of calcium propionate in hypercholesterolemic patients—with serum LDL > 115 mg/dL—is scheduled for completion on 30 November 2020.

A more robust feature of propionate is its influence on satiety. Propionate plays a role in appetite regulation: the administration of inulin-propionate ester was able to reduce food intake of almost 15% in overweight (BMI ~ 25) subjects, over a period of 24 weeks [265]. Other beneficial effects of the treatment included the direct stimulation of colonic cells to produce PYY and GLP-1, which had positive effects on glucose tolerance and overall energy intake.

The increase in colonic propionate is indeed a desired goal for appetite modulation but has major drawbacks. Orally administered SCFA display very low palatability, and they are readily absorbed in the small intestine, where L cells, responsible for the synthesis of both PYY and GLP-1 [266], are rare. As for the case of propionate, diets with fiber supplements do not predictably or reliably increase colonic production or circulating levels of propionate in humans because of the inherent variability in gut microbial makeup [265]. Appropriate formulations, such as the aforementioned inulin-propionate ester, should be used instead.

Acetate—the most abundantly produced SCFA—is efficiently absorbed in the colon [267], and is made available to peripheral tissues, where it can either be used for lipogenesis, or immediately oxidized for energy production [143]. Even though acetate is a ubiquitous molecule, pivotal to most metabolic pathways, it has nevertheless been shown how acetate supplementation in a high-fiber diet is able to reduce blood pressure [268], and stable isotope-labeled colonic acetate has been shown to reach the central nervous system, resulting in hypothalamic neuronal activation ultimately leading to appetite suppression (Figure 3) [269].

All the single evidences pointing towards overall beneficial effects of SCFA notwithstanding, the integration of all individual evidences—each one thoroughly dissected in separate experiments often performed in animal models—into a coherent picture, remains elusive. SCFA are themselves a source of energy, contributing to lipid and glucose synthesis, and some epidemiological studies [270,271] and—to some extent—mouse studies [35], have indicated a positive association between higher fecal SCFA concentrations and body weight. These investigations, performed on a limited number of subjects, were confirmed in a recent study on 441 men and women, where—after adjusting for confounders—fecal SCFA levels were associated with increases in adiposity (increased BMI, body fat and waist circumference) and cardiometabolic risk indicators (increased VLDL and triglycerides, reduced HDL, increased systolic, diastolic and mean blood pressure) [272]. The absence of data on circulating, and thus bioactive, SCFA does not allow us to draw clear conclusions. However, given the countless combinations of human genetics (the other major determinant, along with the diet, of the microbiota composition) and microbiota makeup, particular care should be taken when trying to translate clear-cut results from in vitro and animal studies into the human setting. The topic, while blooming at a fast rate, is still in its infancy, and personalized treatments, as already discussed, may prove themselves the best approach in the long run.

### 3.4. Choline Metabolites and CVD

In 2011, a work by Hazen and Lusis stirred the scientific community by finding, through an unbiased small-molecule metabolomics approach, that choline, and the products of its catabolism—produced by gut microbiota—promoted atherosclerosis development [273]. Intestinal microbes metabolize choline into trimethylamine (TMA), a gas at body temperature, which is further metabolized by the hepatic flavin mono-oxygenases (FMOs), especially by FMO3, into trimethylamine oxide (TMAO) [274,275]. They demonstrated how atherosclerosis in ApoE^-/-^ mice correlated in a dose-dependent manner with increasing levels of dietary choline and TMAO. This effect was completely abolished by the suppression of the intestinal flora using an antibiotic cocktail. A potential mechanism might involve macrophages: two scavenger receptors implicated in atherosclerosis—CD36 and SR-A1—were upregulated in macrophages from mice fed with diets supplemented with either choline or TMAO. Once again, treatment with antibiotics did not result in the upregulation of the same genes [273]. Later on, it was found that TMAO also promotes vascular inflammation by eliciting the NF-κB signaling cascade in both human aortic endothelial cells, as well as vascular smooth muscle cells [276]. Furthermore, TMAO-exposed endothelial cells were more efficient in promoting the recruitment of activated lymphocytes.

Reportedly, red meat—whose consumption is associated with CVD—is a potential source of TMAO, and experiments performed in healthy volunteers traced an increase in TMAO upon meat consumption from an increased microbial metabolism of carnitine (but not choline), as well as from reduced renal TMAO excretion [277]. This fits nicely with the previously described picture, but it must be taken into account that fish and its related products, while correlated with a reduced atherosclerosis risk [278,279], are a major choline source [280]. Fish might contain elevated choline levels, yet that is balanced by omega-3 [281] and anti-atherogenic peptide content [278], whereas red meat consumption is purportedly athero-prone for its high-salt, saturated fat content and polycyclic aromatic carcinogens caused by high-temperature cooking [282]. It must be taken into account how these aspects are not necessarily related to red meat.

Over the years it has been demonstrated that TMAO not only contributes to atherosclerosis [283,284,285,286,287,288], but also to heart failure [289,290], thrombosis [291], cardiac transplant outcome [292], and chronic kidney disease [293,294]. However, it is important to note that several dietary trials did not find an association between the intake of choline-rich food—or circulating TMAO levels—and CVD risk [32,295,296]. To further complicate the matter, a study demonstrated that the administration of L-carnitine to male ApoE^-/-^ mice transfected with human cholesteryl ester transfer protein (hCETP) results in a significant increase in plasma TMAO levels that were surprisingly inversely correlated with aortic lesion size in both aortic root and thoracic aorta [297].

Additionally, two studies testing the effect of dietary choline in ApoE^-/-^ mice [190] and both Ldlr^-/-^ and ApoE^-/-^ mice [298] concluded that there was no correlation between a choline supplemented diet and atherosclerosis lesion size. However, in both studies the size of the aortic lesions was not provided in absolute values, but it was normalized to the vessel area. This is an approach widely used for vessel analysis in vivo [299,300,301,302,303,304], but not in histology, where post-mortem modifications may occur, introducing experimental errors [305]. Interestingly, absolute lesion values, provided in the supplementary information of one of the two studies [190], showed an increased lesion size in mice fed more choline.

Trying to reconcile all the above data, it can be hypothesized that TMAO plays a role in atherosclerosis, although not dramatic, and such effect may be masked, above all, by genetic and dietary factors (Figure 3).

## 4. Conclusions

While the field of research into microbiota is still developing, accumulating data from an increasing number of studies have highlighted an important link between gut microbiota and host pathophysiology. Gut microbiota is strictly connected to the endocrine system, through the secretion of hormones, the modulation of hormone release by the host, and the conversion of dietary and endogenous metabolites into molecules with hormone-like properties that allow for the communication with peripheral organs and tissues in the host. 

The microbiota can thus influence a broad range of physiological processes, including host behavior, appetite, energy metabolism and the immune response. As a consequence, alterations in the distribution of the species that make up the intestinal microbiota (dysbiosis) can modify the plasma concentrations of such molecules, resulting into a plethora of pathological states. In particular, dysbiosis and altered plasma concentrations of primary and secondary bile acids, SCFA and TMAO have been linked to pathological conditions that increase the global CVD risk, such as atherosclerosis, hypertension, heart failure, chronic kidney disease, obesity, and type 2 diabetes.

Most of the results obtained so far and discussed in this review were obtained in rodents; thus, future clinical studies will be necessary to assess the translatability of all these experimental and sometimes conflicting results to the human setting. Nevertheless, the ongoing process of identifying other microbiota-derived metabolites—and the modulation of gut microbiota composition and function through dietary and pharmacological interventions, in order to tweak its endocrine potential—could thus represent an exciting, novel opportunity to improve cardiovascular health and risk prevention.

## Figures and Tables

**Table 1:** The human bile acids and their receptors.

Human Bile Acid Pool	
**Primary BAs**	CA, CDCA
Secondary BAs	LCA, DCA, UDCA, iso-BAs, allo-BAs, keto-BAs
**Receptor**	**Affinity**
FXR	CDCA > DCA > LCA > CA > UDCA
TGR5	LCA > DCA > CDCA > CA > UDCA
PXR	LCA
VDR	LCA
S1PR2	Conjugated BAs (T-CA, G-CA, T-DCA, G-DCA, T-UDCA)

CA: cholic acid; CDCA: chenodeoxycholic acid; LCA: lithocholic acid; DCA: deoxycholic acid; UDCA: ursodeoxycholic acid; T-: taurine conjugated; G: glycine conjugated.
